# Sarin exposure, mortality and cancer incidence in UK military veterans involved in human experiments at Porton Down: 52-year follow-up

**DOI:** 10.1136/oemed-2024-109525

**Published:** 2024-09-30

**Authors:** Gemma Archer, Thomas Keegan, Simon Wessely, Katherine M Venables, Nicola T Fear

**Affiliations:** 1 Department of Medical Education, Brighton and Sussex Medical School, Brighton, UK; 2 King’s Centre for Military Health Research, Department of Psychological Medicine, King's College London, London, UK; 3 Lancaster Medical School, Lancaster University, Lancaster, UK; 4 Nuffield Department of Population Health, Oxford University, Oxford, UK

**Keywords:** Epidemiology, Military Personnel, Mortality, Materials, exposures or occupational groups

## Abstract

**Objectives:**

We investigated whether UK military personnel exposed to sarin during the ‘Service Volunteer Programme’ at Porton Down had increased rates of mortality or cancer incidence over a 52-year follow-up.

**Methods:**

A historical cohort study assembled from UK military records, comprising male veterans exposed to sarin during the ‘Service Volunteer Programme’ at Porton Down, UK (n=2975) and a comparison group of similar veterans who did not attend (n=2919). Mortality and cancer incidence data were obtained from national registries up to December 2019. Analysis was conducted using Cox regression adjusted for age, year of birth and service characteristics.

**Results:**

Over a median follow-up of 52.2 years (range 2 days to 74.6 years), 1598 (53.7%) sarin-exposed veterans and 1583 (54.3%) non-exposed veterans died. Adjusted HRs for all-cause mortality were raised for any sarin exposure (HR=1.08, 95% CI 1.01 to 1.16), two or more exposures (HR=1.25, 95% CI 1.04 to 1.49) and higher doses (air >15 mg.min/m^3^) (HR=1.15, 95% CI 1.02 to 1.30). For cause-specific mortality, sarin exposure was associated with deaths from ‘other’ circulatory diseases (excludes ischaemic and cerebrovascular diseases) (HR=1.41, 95% CI 1.06 to 1.87) and alcohol-attributable deaths (HR=2.66, 95% CI 1.40 to 5.07). There was no association between sarin exposure and overall cancer incidence (HR=1.01, 95% CI 0.93 to 1.10), but cancer incidence was higher for alcohol-related neoplasms (HR=1.24, 95% CI 1.01 to 1.51).

**Conclusions:**

Sarin exposure was associated with increased rates of mortality over a 50-year follow-up. The strongest associations were observed for deaths attributable to alcohol and ‘other’ circulatory diseases.

**WHAT IS ALREADY KNOWN ON THIS TOPIC**

- The nerve agent sarin’s acute effects are well understood, but little is known about its long-term effects on health, especially the effects of low-dose exposures in military and civilian populations.

**WHAT THIS STUDY ADDS**

- This study of UK military veterans presents a novel examination of the relationship between sarin exposure and long-term health due to its unusually detailed data on sarin exposure and long follow-up. Sarin exposure was associated with an increase in all-cause mortality, with higher rates observed for those exposed to two or more tests and tests with higher doses, compared with unexposed veterans. Higher mortality rates were also found for deaths from circulatory diseases and alcohol-related deaths. There was less evidence of an association between sarin exposure and cancer incidence.

**HOW THIS STUDY MIGHT AFFECT RESEARCH, PRACTICE OR POLICY**

- Our findings show that a small number of sarin-exposed veterans had higher rates of specific causes of death, which may warrant further investigation. It is not possible for us to attribute our findings causally to the effects of sarin, but health providers should be aware of potential concerns in occupational and civilian populations at risk of potential exposure.

## Introduction

 Sarin (GB or 2-(fluoro(methyl)phosphoryl]oxypropane) is a highly potent organophosphate nerve agent that acts by inhibiting acetylcholinesterase activity within the central, peripheral and autonomic nervous systems. Sarin’s acute effects are well understood. They include muscle twitching, rapid breathing, chest tightness, changes in heart rate, nausea and abdominal pain, with large doses leading to paralysis, unconsciousness and death. Less is known about its long-term effects on health, especially the effects of low-dose exposures in military and civilian populations^1^ (ie, doses at a level sufficient to generate the signs and symptoms of sarin exposure).

A review by the US National Toxicology Programme found moderate evidence of morphological and histological changes in human nervous system tissue in the years following acute sarin exposure among individuals who might have been exposed during military operations and victims of the Tokyo subway attack.^1^ However, it has not been possible to draw conclusions regarding other health outcomes due to a scarcity of quality studies, with most lacking accurate exposure information, relying on self-reported outcomes, unsuitable comparison groups or small sample sizes.^1 2^

Sarin has also recently been implicated as a possible cause of what is often called ‘Gulf War syndrome’,^3^ better understood as ‘Gulf War illnesses’.^4^ Some have argued that exposure to sarin, either during or immediately after the 1991–1992 Gulf War, caused long-term multisymptom ill health in some veterans, but this is strongly contested.^5^ A key issue that prevents any conclusions being drawn about the role of sarin in Gulf War illnesses is that sarin exposure has only been assessed by proxy, for example, mapping of a possible ‘sarin plume’ from the destruction of the Iraq chemical weapon arsenal after the war,^2^ or by retrospective self-report,^6^ which is subject to recall bias and reverse causality.

In this paper, we report the long-term outcomes of a historical cohort in which acute exposure to sarin was documented in detail.^7 8^ We examine whether approximately 3000 veterans exposed to sarin as part of the ‘Service Volunteer Programme’ at Porton Down, UK, had increased rates of mortality or cancer incidence over an average of 52 years of follow-up.

## Methods

### The Porton Down veterans cohort study

The Porton Down veterans cohort study was established in 2001 in response to concerns about the long-term health of former service personnel involved in chemical experiments between 1941 and 1989 as part of the ‘Service Volunteer Programme’ at the UK government research establishment, Porton Down.^7^ The Porton Down Veterans Cohort Study is a historical cohort study that comprises two groups of military veterans: the ‘Porton Down veterans’—all those identified from historical records at Porton Down as having participated in the Service Volunteer Programme between 1 April 1941 and 31 December 1989—and ‘non-Porton Down veterans’—a comparison cohort of veterans who did not attend Porton Down and who were identified by selecting the adjacent service number (alternately above and below) of every Porton Down veteran.^9 10^

### Chemical exposure

Information on chemical exposures was assembled from experimental records in the Porton Down historical archive. The number of veterans identified as undergoing tests was consistent with other Porton Down documents.^11^ A total of 492 chemicals were identified and, where possible, assigned to North Atlantic Treaty Organisation chemical warfare agent categories.^12^ Other categories included, for example, corrosives, pesticides, detergents, known carcinogens and non-chemical tests.^13^ For all chemical tests, the date of exposure and chemical name were abstracted.^12^ For sarin, additional information was collected on dose (exposure quantity) used in chamber tests (high (≥15 mg.min/m^3^), low (<15 mg.min/m^3^ or unknown), exposure route (eg, dermal, inhalation, intramuscular and intravenous) and the presence or not of any physical protection (eg, clothing or respirator) or chemical protection (eg, barrier creams or nerve agent prophylactics such as pyridostigmine). Further detail on the abstraction of chemical exposure data can be found elsewhere.^8 11^

### Mortality and cancers

Information on mortality and emigration was obtained primarily from the National Health Service Central Register, with some deaths identified from the Department for Work and Pensions and military personnel records (n=102; 3.2%).

Cancer site and date of registration were available from national cancer registries as of 1 January 1971. We also, from 1971, included cancers listed on death certificates if they were reported as an underlying cause of death; for these cancers, we used the date of death as the date of cancer registration (n=51, 2.5%).

Cause of death and cancer registrations were coded according to International Classification of Diseases, 10th revision (ICD-10).^14^ We focused on the most common causes of death and types of cancer; other outcomes of interest were identified a priori based on plausible mechanisms and associations demonstrated in existing chemical warfare literature; these included deaths from infectious and parasitic diseases,^9^ brain and other central nervous system neoplasms,^2 15^ genitourinary diseases,^9^ circulatory diseases,^1 2 9^ deaths from external causes^9^ and ill-defined and in situ neoplasms and neoplasms of uncertain or unknown behaviour.^10^ All other outcomes were exploratory.

Deaths 100% attributable to alcohol were defined by ICD-10 codes F102, K701, K703, K704 and K709^16^ and deaths strongly causally related to smoking by ICD-10 codes C33, C34 and J40–J44.^17^ Alcohol-related cancers were defined by ICD-10 codes C01–C06, C09–C10, C12–C13, C15, C18, C19–C20, C22, C32 and C50^16^ and smoking-related cancers by C00–C16, C18–C20, C22, C25, C32–C34, C53, C64–C67 and C92.^17^

### Follow-up

For mortality, person-years of follow-up for Porton Down veterans started from the date of their first test at Porton Down (or date of arrival if missing). Follow-up for mortality was until the date of death or 31 December 2019, or censored at the last known date alive (ie, date of emigration, discharge from the services or end of the first phase of follow-up if previously traced).

For cancer analysis, person-years of follow-up for Porton Down veterans started from 1 January 1971 or the date of the first test (or arrival) at Porton Down, whichever came later. Follow-up for cancer was until the date of first cancer registration or until 31 December 2019, or censored at death or date last known alive.

The start of follow-up for non-Porton Down veterans was derived by adding to the enlistment date the interval between the corresponding Porton Down veterans’ dates of enlistment and the start of follow-up.

### Potential confounders

Confounders were factors possibly associated with sarin exposure and mortality or cancer incidence; these included age at exposure/start of follow-up (years), year of birth, service at enlistment (Royal Navy and Marines, Army and Royal Air Force), previous duration of service (years), period of enlistment (before or during World War II, after World War II and post-National Service), place of birth (England, Scotland, Wales, Northern Ireland and others) and co-exposure to other chemical agents, with potentially harmful effects.^11^

### Analysis sample

Of all valid records retrieved, 18 441 (95.9%) Porton Down veterans and 17 846 (92.8%) non-Porton Down veterans were successfully traced or data were available from the original first phase of data collection^7^ (online supplemental figure 1). Exclusions included those with missing or implausible data (n=563; eg, veterans who were aged <14 years at enlistment, veterans who died prior to their date of enlistment), women (n=132; exposure data were not available for women as it was not abstracted from experiment books during the original assembly of the cohort) and Porton Down veterans who were not exposed to sarin (n=15 158). Cohort members who were eligible for inclusion in these analyses were all UK servicemen who were exposed to sarin during the Service Volunteer Programme at Porton Down between 1 April 1941 and 31 December 1989, and their corresponding non-Porton Down veteran, who had complete data on mortality, start and end-person years of follow-up and confounders. The analytical sample for mortality consisted of 2975 Porton Down veterans and 2919 corresponding non-Porton Down veterans.

Prior to the start of follow-up for cancers (1 January 1971), 51 Porton Down and 44 non-Porton Down veterans had died, and 25 Porton Down and 22 non-Porton Down veterans were lost to follow-up. The analytical sample for cancer incidence therefore consisted of 2899 Porton Down veterans and 2853 non-Porton Down veterans.

### Statistical analysis

Cox regression proportional hazards models were used to compare mortality rates between sarin-exposed Porton Down veterans and non-Porton Down veterans, with age since start-of-follow-up as the time scale. First, we conducted exploratory analyses examining all-cause mortality rates by sarin exposure (any), number of sarin tests, sarin dose and sarin tests with and without chemical or physical modifiers. Models were adjusted for demographics: age (age at the start of follow-up) and calendar period (year of birth); baseline hazard functions for calendar period were allowed to vary by stratum (5-year bands). Models were then further adjusted for military service characteristics (branch of service, previous duration of service, period of joining and place of birth).

To account for chemical co-exposure, we excluded veterans exposed to any potentially harmful chemical other than sarin, that is, sarin-only exposure. To maintain our sample size, we permitted co-exposure to two commonly used chemicals deemed unlikely to cause long-term harm: rubber mixes (n=1697) and ‘sulphur mustard sensitivity tests’ (a low dose exposure used prior to ‘full’ sulphur mustard testing; n=992). In sensitivity analysis, mortality HRs for sarin-only exposure were similar, including or excluding co-exposure to rubber mixes and sulphur mustard sensitivity tests (online supplemental table 1).

Next, we assessed associations between sarin exposure (‘any’ and ‘only’) and cause-specific mortality. Since associations were potentially modified by the presence of physical protection, in post hoc analyses, we also examined associations between sarin-only exposure and mortality restricted to veterans who received no physical protection (n=2292).

Experimental programmes at Porton Down changed over time therefore we also tested interactions between the period of attendance at Porton Down and mortality rates using likelihood ratio tests. The proportional hazards assumption was tested using Schoenfeld residuals.

The above analyses were repeated for cancer incidence outcomes as appropriate.

All analyses were conducted using STATA V.16.1^18^

## Results

### Descriptive characteristics

Descriptive characteristics for sarin-exposed Porton Down veterans and non-Porton Down veterans were largely identical across most demographic and military factors (table 1). For both groups of veterans, the majority were born in England in the 1930s and enlisted after World War II, although sarin-exposed veterans appeared to serve for longer; for example, 29.3% of sarin-exposed veterans served over 10 years compared with 19.8% of non-Porton Down veterans.

**Table 1:** Characteristics of 2975 Porton Down veterans exposed to sarin and 2919 non-Porton Down veterans

	Sarin-exposed Porton Down veterans (n=2975)	Non-Porton Down veterans (n=2919)
	n (%)	n (%)
Service at enlistment		
Army	1192 (40.1)	1165 (39.9)
Airforce	1082 (36.4)	1074 (36.8)
Navy	701 (23.6)	680 (23.3)
Decade of birth		
<1920	19 (0.6)	41 (1.4)
1920–1929	292 (9.8)	311 (10.7)
1930–1939	1993 (67.0)	1933 (66.2)
>1940	671 (22.6)	634 (21.7)
Place of birth		
England	2440 (82.0)	2338 (80.1)
Scotland	264 (8.9)	298 (10.2)
Wales	121 (4.1)	133 (4.6)
Northern Ireland	41 (1.4)	41 (1.4)
Other	109 (3.7)	109 (3.7)
Age at enlistment (years)		
<16	238 (8.0)	216 (7.4)
16–17	930 (31.3)	825 (28.3)
18–19	1353 (45.5)	1303 (44.6)
20–21	336 (11.3)	378 (12.9)
>21	118 (4.0)	197 (6.7)
Age at start of follow-up (years)		
14 to <20	1257 (42.3)	1191 (40.8)
20 to <22	835 (28.1)	794 (27.2)
22 to <25	618 (20.8)	599 (20.5)
25 to <35	242 (8.1)	298 (10.2)
35+	23 (0.8)	37 (1.3)
Period of enlistment*		
Before or during World War II	94 (3.1)	104 (3.6)
After World War II	2339 (78.6)	2295 (78.6)
Post-National Service	542 (18.2)	520 (17.8)
Rank at enlistment		
Private	2952 (99.6)	2901 (99.6)
Other	13 (0.4)	12 (0.4)
Missing	10	6
Total duration of service (years)		
<2	107 (3.6)	450 (15.4)
2<3	831 (28.0)	950 (32.5)
3<5	318 (10.7)	330 (11.3)
5<10	842 (28.4)	610 (20.9)
10+	870 (29.3)	579 (19.8)
Missing	7	0
Previous duration of service before test/start of follow-up (years)		
<1	577 (19.4)	573 (19.6)
1<2	995 (33.4)	982 (33.6)
2<5	922 (31.0)	897 (30.7)
5<10	381 (12.8)	373 (12.8)
10+	100 (3.4)	94 (3.2)
Any neoplasm†		
Yes	1009 (34.8)	1021 (35.8)
No	1890 (65.2)	1832 (64.2)
Total number of cancers†		
0	1890 (65.2)	1832 (64.2)
1	789 (27.2)	792 (27.8)
2+	220 (7.6)	229 (8.0)
Vital status		
Deceased	1598 (53.7)	1583 (54.2)
Alive	1042 (35.0)	1032 (35.4)
Lost to follow-up‡	335 (11.3)	304 (10.4)

* World War II dates taken as 1 September 1939–30 April 1945; the period when national service was in force was taken as 1 May 1945–31 December 1960, with the sample, including both regulars and National Service personnel.

† Of 2899 Porton Down veterans and 2853 non-Porton Down veterans alive at the start of follow-up for cancer (1 January 1971).

‡ Last known date alive in the UK, for example, discharge from the services, emigration or date last traced.

Most sarin-exposed veterans attended Porton Down during the 1950s (72.8%) and the median age at attendance was 20.3 (range 16–44) (table 2). Veterans underwent a single sarin test (93.5%), with approximately a quarter of tests involving physical or chemical protective equipment. Veterans were often co-exposed to other chemicals, most commonly sulphur mustard (32.1%) and atropine (12.1%), with 48.1% of veterans exposed to sarin only.

**Table 2:** Characteristics of sarin exposure in 2975 Porton Down veterans

	Sarin-exposed Porton Down veterans (n=2975)
	n (%)
Decade of first test at Porton Down	
1940s	71 (2.4)
1950s	2166 (72.8)
1960s	363 (12.2)
1970s	266 (8.9)
1980s	109 (3.7)
Number of sarin tests	
1	2783 (93.5)
2+	192 (6.5)
Sarin exposure quantity (dose)*	
Unknown	492 (19.0)
Low (air<15 mg.min/m^3^)	1455 (55.4)
High (air>15 mg.min/m^3^)	657 (25.5)
Protective equipment†	
Physical	683 (23.0)
Chemical	731 (24.6)
State	
Vapour	2577 (86.7)
Liquid	349 (11.7)
Not recorded	47 (1.6)
Chemical co-exposure	
Other nerve agents	11 (0.4)
Sulphur mustard	954 (32.1)
CS (2-chlorobenzylidene malononitrile)‡	215 (7.2)
CR (dibenzoxazepine)	238 (8.0)
Pralidoxime	175 (5.9)
Atropine	364 (12.2)
Pyridostigmine	23 (0.8)
Sarin-only exposed‡	1430 (48.1%)

* Chamber tests only.

† Both physical and chemical protection recorded for 167 veterans.

‡ Allows for co-exposure to rubber mixes and sulphur mustard sensitivity tests.

### Mortality

1598 sarin-exposed Porton Down veterans and 1583 non-Porton Down veterans died over a median follow-up of 52.2 years (range 2 days to 74.6 years). Incidence rates for all-cause mortality were 10.8 per 1000 person-years for Porton Down veterans and 10.7 per 1000 person-years for non-Porton Down veterans.

In fully adjusted models (models adjusted for demographics and military service characteristics), exposure to any sarin was associated with a small increase in all-cause mortality (HR=1.08, 95% CI 1.01 to 1.16) compared with non-Porton Down veterans (table 3). HRs were raised for veterans who were involved in two or more tests (n=220; HR=1.25, 95% CI 1.04 to 1.49), as opposed to a single test (n=2783; HR=1.06, 95% CI 0.99 to 1.14). Likewise, veterans exposed to ‘high’ doses (HR=1.15, 95% CI 1.02 to 1.30) had slightly larger HRs compared with those exposed to ‘low’ doses (HR=1.04, 95% CI 0.95 to 1.13), compared with non-Porton Down veterans. The presence of physical protective equipment appeared to attenuate associations with all-cause mortality (HR=1.00, 95% CI 0.90 to 1.12), but this attenuation was not evident for tests that involved ‘chemical protection’ (HR=1.10, 95% CI 0.93 to 1.29). Associations between sarin exposures and all-cause mortality were largely similar in models restricted to sarin-only exposures—where co-exposures to other potentially harmful chemicals were excluded from analyses (table 3).

**Table 3:** Fully adjusted HRs for the association between sarin exposure and all-cause mortality and any cancer incidence

	HR (95% CI) all-cause mortality		HR (95% CI) for cancer incidence	
	Fully adjusted*	Fully adjusted*sarin only exposed†	Fully adjusted*	Fully adjusted*sarin only exposed‡
Sarin exposure				
(yes/no)	1.08 (1.01 to 1.16)	1.08 (1.00 to 1.18)	1.01 (0.93 to 1.10)	1.05 (0.94 to 1.17)
Number of sarin tests				
1	1.06 (0.99 to 1.14)	1.07 (0.98 to 1.17)	1.00 (0.92 to 1.10)	1.04 (0.93 to 1.16)
2 or more	1.25 (1.04 to 1.49)	1.26 (1.00 to 1.59)	1.09 (0.85 to 1.38)	1.04 (0.76 to 1.43)
Dose				
Unknown	1.10 (0.99 to 1.22)	1.13 (1.01 to 1.27)	1.03 (0.91 to 1.17)	1.06 (0.91 to 1.24)
Low (air<15 mg.min/m^3^)	1.04 (0.95 to 1.13)	1.03 (0.92 to 1.15)	0.99 (0.89 to 1.11)	1.01 (0.88 to 1.16)
High (air>15 mg.min/m^3^)	1.15 (1.02 to 1.30)	1.15 (0.94 to 1.41)	1.01 (0.87 to 1.18)	1.07 (0.83 to 1.38)
State§				
Vapour	1.07 (1.00 to 1.15)	1.08 (0.98 to 1.18)	1.01 (0.92 to 1.10)	1.05 (0.93 to 1.18)
Liquid	1.10 (0.96 to 1.27)	1.11 (0.96 to 1.29)	1.02 (0.85 to 1.23)	1.01 (0.83 to 1.23)
Physical protection				
No	1.11 (1.03 to 1.20)	1.11 (1.01 to 1.23)	1.02 (0.93 to 1.13)	1.12 (0.99 to 1.26)
Yes	1.00 (0.90 to 1.12)	1.03 (0.91 to1.17)	0.96 (0.84 to 1.11)	0.89 (0.75 to 1.06)
Chemical protection				
No	1.06 (0.99 to 1.15)	1.08 (0.99 to 1.19)	1.01 (0.92 to 1.11)	1.04 (0.92 to 1.17)
Yes	1.13 (1.01 to 1.26)	1.10 (0.93 to 1.29)	1.01 (0.88 to 1.17)	1.05 (0.85 to 1.29)

All-cause mortality models based on 2975 sarin-exposed veterans and 2,919 ‘Non-Porton Down veterans’ (reference); over an average 52.2-year follow-up. Cancer incidence models based on 2899 sarin-exposed veterans, and 2,853 ‘Non-Porton Down veterans’ (reference);, over an average 49.8-year follow-up

* Adjusted for age, calendar period, branch of service, previous duration of service and place of birth.

† Estimates based on 1430 veterans exposed to sarin only (allowing for co-exposure to rubber mixes and mustard sensitivity tests).

‡ Estimates based on 1385 veterans exposed to sarin only (allowing for co-exposure to rubber mixes and mustard sensitivity tests).

§ Excludes 47 veterans with missing state data.

### Cause-specific mortality

For cause-specific mortality, sarin exposure (any) was associated with raised mortality rates in fully-adjusted models for ‘all other circulatory’ diseases (HR=1.41, 95% CI 1.06 to 1.87), diseases of the digestive system (HR=1.42, 95% CI 1.00 to 2.03), alcohol-attributable deaths (HR=2.66, 95% CI 1.40 to 5.07) and missing cause of death (HR=1.57, 95% CI 1.04 to 2.35), compared with non-Porton Down veterans (table 4). The exclusions of veterans co-exposed to other chemicals and whose tests included physical protection strengthened associations across several causes of death, namely, deaths from malignant neoplasms (any) (HR=1.24, 95% CI 1.06 to 1.46), lung cancers (HR=1.39, 95% CI 0.99 to 1.76), intestine and rectum cancers (HR=1.77, 95% CI 1.08 to 2.90), ‘all other’ circulatory diseases (HR=1.76, 95% CI 1.22 to 2.52) and alcohol-attributable deaths (HR=2.66, 95% CI 1.18 to 5.99), but weakened associations for deaths from diseases of the digestive system (HR=1.42, 95% CI 0.89 to 2.28) and missing cause of death (HR=1.31, 95% CI 0.74 to 2.33). There was little evidence of an association between sarin exposure and all other causes of death.

**Table 4:** HRs for the association between attendance at Porton Down and cause-specific mortality in 2975 Porton Down veterans and 2919 non-Porton Down veterans

	Observed deaths (n)		HR (95% CI)			
			**Any sarin exposure**		Sarin-only exposure*	
**Cause of death (ICD-10 code)**	**Porton Down veterans**	**Non-Porton Down veterans**	**Adjusted for age and calendar period**	**Fully adjusted†**	**Fully adjusted†**	**Fully adjusted†—excluding physical protection**
All-cause	1598	1583	1.08 (1.01 to 1.16)	1.08 (1.01 to 1.16)	1.08 (1.00 to 1.18)	1.12 (1.01 to 1.23)
Infectious and parasitic (A00–B99)	17	10	1.88 (0.86 to 4.12)	1.97 (0.89 to 4.36)	1.55 (0.60 to 4.05)	0.91 (0.25 to 3.35)
Malignant neoplasms (All) (C00–C97)	543	534	1.08 (0.96 to 1.22)	1.07 (0.95 to 1.21)	1.14 (0.98 to 1.31)	1.24 (1.06 to 1.46)
Upper aerodigestive (C00–C14 and C30–C32)	20	17	1.21 (0.63 to 2.32)	1.20 (0.63 to 2.29)	1.13 (0.49 to 2.58)	1.59 (0.70 to 3.59)
Oesophageal (C15)	37	37	1.05 (0.66 to 1.65)	1.03 (0.65 to 1.62)	1.05 (0.60 to 1.86)	1.00 (0.52 to 1.92)
Intestine and rectum (C17–C20)	55	45	1.29 (0.87 to 1.92)	1.30 (0.87 to 1.93)	1.61 (1.03 to 2.52)	1.77 (1.08 to 2.90)
Trachea, bronchus and lung (C33 and C34)	159	157	1.07 (0.86 to 1.34)	1.06 (0.85 to 1.32)	1.08 (0.82 to 1.41)	1.32 (0.99 to 1.76)
Brain and other central nervous system (C71 and C72)	16	15	1.09 (0.54 to 2.21)	1.08 (0.53 to 2.19)	1.54 (0.65 to 3.62)	1.69 (0.68 to 4.21)
All lymphatic and haematopoietic (C81–C96)	44	44	1.05 (0.69 to 1.60)	1.04 (0.69 to 1.58)	1.00 (0.61 to 1.65)	1.04 (0.58 to 1.84)
Nervous system (G00–G99)	49	68	0.77 (0.53 to 1.12)	0.78 (0.54 to 1.13)	0.72 (0.44 to 1.16)	0.62 (0.34 to 1.13)
Circulatory system all (I00–I99)	540	536	1.08 (0.96 to 1.22)	1.08 (0.95 to 1.21)	1.10 (0.95 to 1.27)	1.13 (0.96 to 1.33)
Ischaemic heart diseases (I20–I25)	345	343	1.07 (0.92 to 1.24)	1.06 (0.91 to 1.23)	1.03 (0.86 to 1.24)	1.04 (0.84 to 1.28)
Cerebrovascular diseases (I60–I69)	87	108	0.89 (0.67 to 1.19)	0.88 (0.66 to 1.17)	1.00 (0.71 to 1.39)	0.97 (0.66 to 1.43)
All other circulatory (I00–I19, I26–I59 and I70–I99)	108	85	1.38 (1.03 to 1.83)	1.41 (1.06 to 1.87)	1.49 (1.08 to 2.08)	1.76 (1.22 to 2.52)
Respiratory system all (J00–J99)	148	158	1.05 (0.83 to 1.31)	1.05 (0.84 to 1.32)	0.97 (0.73 to 1.28)	0.84 (0.59 to 1.18)
Chronic lower respiratory tract (J40–J47)	81	86	1.03 (0.76 to 1.40)	1.03 (0.76 to 1.40)	1.04 (0.72 to 1.50)	0.86 (0.54 to 1.35)
All other respiratory (J00–39 and J48–99)	67	72	1.07 (0.76 to 1.49)	1.07 (0.76 to 1.50)	0.87 (0.57 to 1.35)	0.80 (0.47 to 1.35)
Digestive system (K00–K93)	72	53	1.42 (0.99 to 2.02)	1.42 (1.00 to 2.03)	1.34 (0.88 to 2.04)	1.42 (0.89 to 2.28)
Genitourinary system (N00–N99)	21	18	1.23 (0.65 to 2.31)	1.25 (0.67 to 2.35)	0.82 (0.36 to 1.84)	0.92 (0.36 to 2.33)
All external causes (S00–T98 and V01–Y98)	75	69	1.10 (0.79 to 1.53)	1.10 (0.79 to 1.52)	1.12 (0.74 to 1.70)	1.14 (0.71 to 1.83)
Alcohol attributable (F102, K701, K703, K704 and K709)	33	13	2.60 (1.37 to 4.94)	2.66 (1.40 to 5.07)	2.12 (0.98 to 4.58)	2.66 (1.18 to 5.99)
Smoking-related (highly causal only; C33, C34 and J40–J44)	237	240	1.06 (0.88 to 1.27)	1.05 (0.87 to 1.25)	1.07 (0.86 to 1.33)	1.16 (0.91 to 1.48)
No ICD code	58	40	1.54 (1.03 to 2.31)	1.57 (1.04 to 2.35)	1.38 (0.85 to 2.25)	1.31 (0.74 to 2.33)

Reference category: ‘Nnon-Porton Down veterans’

* 1430 Porton Down veterans exposed to sarin only (allowing for co-exposure to rubber mixes and mustard sensitivity tests).

† Adjusted for branch of service, previous duration of service, period of joining and place of birth.

ICD-10International Classification of Diseases, 10th revision

### Cancer incidence

1009 (34.8%) sarin-exposed Porton Down veterans and 1021 (35.8%) non-Porton Down veterans had at least one cancer over a median follow-up of 49.8 years (range of 70 days to 74.6 years) (table 1). Cancer incidence rates were almost identical: 9.7 per 1000 person-years for sarin-exposed veterans and 9.9 per 1000 person-years for non-Porton Down veterans.

Table 3 shows little evidence of an association between sarin exposure and overall cancer incidence. For example, in fully adjusted models, exposure to any sarin (HR=1.01, 95% CI 0.93 to 1.10), two or more tests (HR=1.09, 95% CI 0.85 to 1.38) or a ‘high’ dose (HR=1.01, 95% CI 0.87 to 1.18) was not associated with cancer incidence, compared with non-Porton Down veterans.

For the type of cancer (table 5), there were no associations between sarin exposure and almost all types of cancer in fully adjusted models, with excess mortality only observed for alcohol-related neoplasms (HR=1.24, 95% CI 1.01 to 1.51). Notably, the exclusion of co-exposure to other chemicals and those whose experiments included physical protective equipment strengthened associations for several types of cancer; excess cancer incidence was observed for malignant neoplasms (HR=1.14, 95% CI 1.01 to 1.29), neoplasms of the intestine and rectum (HR=1.42, 95% CI 1.00 to 2.01), lungs (HR=1.40, 95% CI 1.07 to 1.83), alcohol-related (HR=1.41, 95% CI 1.08 to 1.85) and smoking-related neoplasms (HR=1.22, 95% CI 1.03 to 1.45).

**Table 5:** HRs for the association between attendance at Porton Down and cancer incidence in 2899 Porton Down veterans and 2853 non-Porton Down veterans

	Number of cases (n)		HR (95% CI)			
			**Any sarin exposure**		**Sarin-only exposure**	
**Cancer type/site (ICD-10 code)**	**Porton Down veterans**	**Non-Porton Down veterans**	**Adjusted for age and calendar period**	Fully adjusted*	Fully adjusted*	Fully adjusted*—excluding physical protection
Any neoplasm (C00–C97 and D00–D48)	1009	1021	1.01 (0.93 to 1.11)	1.01 (0.93 to 1.10)	1.05 (0.94 to 1.17)	1.13 (1.00 to 1.27)
Any malignant neoplasm (C00–C97)	961	967	1.02 (0.93 to 1.11)	1.02 (0.93 to 1.11)	1.05 (0.94 to 1.17)	1.14 (1.01 to 1.29)
Upper aerodigestive (C00–C14 and C30–C32)	43	34	1.28 (0.82 to 2.01)	1.30 (0.83 to 2.03)	1.16 (0.64 to 2.08)	1.45 (0.78 to 2.68)
Oesophagus (C15)	39	36	1.13 (0.72 to 1.78)	1.12 (0.71 to 1.76)	1.08 (0.60 to 1.93)	1.09 (0.56 to 2.11)
Stomach (C16)	27	35	0.82 (0.50 to 1.36)	0.81 (0.49 to 1.34)	0.82 (0.45 to 1.50)	0.77 (0.37 to 1.61)
Intestine and rectum (C17–C20)	120	106	1.18 (0.91 to 1.54)	1.19 (0.91 to 1.54)	1.35 (0.99 to 1.83)	1.42 (1.00 to 2.01)
Pancreas (C25)	26	21	1.33 (0.75 to 2.36)	1.34 (0.75 to 2.38)	1.03 (0.50 to 2.11)	0.75 (0.28 to 1.99)
Trachea, bronchus and lung (C33 and C34)	179	173	1.10 (0.89 to 1.35)	1.09 (0.89 to 1.35)	1.08 (0.84 to 1.39)	1.40 (1.07 to 1.83)
Melanoma of skin (C43)	22	27	0.84 (0.48 to 1.48)	0.83 (0.47 to 1.45)	0.75 (0.35 to 1.63)	0.76 (0.31 to 1.85)
Other skin (C44)	223	278	0.83 (0.69 to 0.99)	0.82 (0.69 to 0.98)	0.82 (0.66 to 1.03)	0.90 (0.70 to 1.16)
Prostate (C61)	191	187	1.07 (0.87 to 1.30)	1.06 (0.87 to 1.30)	1.19 (0.93 to 1.53)	1.19 (0.90 to 1.59)
Bladder (C67)	44	53	0.87 (0.58 to 1.30)	0.87 (0.58 to 1.29)	0.96 (0.60 to 1.55)	0.87 (0.49 to 1.56)
Other urinary tract (C64–C66 and C68)	27	37	0.76 (0.46 to 1.24)	0.75 (0.46 to 1.23)	0.92 (0.50 to 1.70)	0.80 (0.38 to 1.67)
Brain and other central nervous system (C71 and C72)	17	17	1.02 (0.52 to 2.01)	1.02 (0.52 to 2.01)	1.36 (0.61 to 3.04)	1.60 (0.68 to 3.74)
All leukaemias (C91–C95)	27	29	0.98 (0.58 to 1.66)	0.98 (0.58 to 1.65)	1.01 (0.54 to 1.88)	1.21 (0.62 to 2.39)
Other lymphatic and haematopoietic (C81–-C90 and C96	47	43	1.12 (0.74 to 1.69)	1.12 (0.74 to 1.69)	0.96 (0.57 to 1.64)	0.95 (0.51 to 1.78)
Ill-defined, secondary or unspecified malignant neoplasms (C76–C80)	34	34	1.08 (0.67 to 1.75)	1.07 (0.66 to 1.73)	1.09 (0.62 to 1.92)	1.02 (0.52 to 2.03)
All other primary malignant neoplasms†	64	55	1.21 (0.84 to 1.74)	1.21 (0.85 to 1.74)	1.31 (0.85 to 2.02)	1.45 (0.90 to 2.32)
Alcohol-related neoplasms‡	211	178	1.24 (1.01 to 1.51)	1.24 (1.01 to1.51)	1.33 (1.04 to 1.69)	1.41 (1.08 to 1.85)
Smoking-related neoplams§	506	489	1.09 (0.96 to 1.23)	1.08 (0.96 to 1.23)	1.11 (0.95 to 1.29)	1.22 (1.03 to 1.45)
Any in situ neoplasm (D00–D09)	52	56	0.97 (0.67 to 1.42)	0.96 (0.66 to 1.40)	1.21 (0.76 to 1.93)	1.04 (0.60 to 1.81)
Any benign neoplasm (D10–D36)	7	11	0.63 (0.24 to 1.62)	0.64 (0.25 to 1.64)	0.99 (0.31 to 3.22)	1.34 (0.42 to 4.28)
Any neoplasm of uncertain or unknown behaviour (D37–D48)	51	38	1.42 (0.93 to 2.17)	1.45 (0.95 to 2.21)	1.23 (0.73 to 2.09)	1.10 (0.59 to 2.05)

Reference category: ‘Nnon-Porton Down veterans’.

* Adjusted for branch of service, previous duration of service, period of joining and place of birth.

† Other primary malignant neoplasms (C21–C24, C26, C7, C38, C40, C41, C45, C47–C50, C53, C54, C56, C60, C62, C63, C69, C70 and C73–C75).

‡ Alcohol-related neoplasms (C01–C06, C09–C10, C12–13, C15, C18, C19–C20, C22, C32 and C50).

§ Smoking-related neoplasms (C00–C16, C18–C20, C22, C25, C32-34, C53, C64–C67 and C92).

ICD-10International Classification of Diseases, 10th revision

Across analyses, the proportional hazards assumption was not violated (p>0.05), and we found no evidence that associations differed by period of attendance (likelihood ratio tests p>0.5).

## Discussion

### Summary of results

We found that sarin exposure was associated with a small (8%) increase in all-cause mortality over an average of 52 years of follow-up among Porton Down veterans, compared with non-Porton Down veterans. Excess mortality was slightly higher in veterans exposed to two or more sarin tests (25%) or ‘higher’ (air>15 mg.min/m^3^) doses (15%). For cause-specific mortality, sarin exposure was associated with an increase in deaths from ‘all other’ circulatory diseases (41%), disease of the digestive system (42%) and alcohol-attributable deaths (166%). For cancer incidence, sarin exposure was associated with an increase in alcohol-related neoplasms only (24%).

In subgroup analyses, veterans who were exposed to sarin only and without any physical protective equipment had raised mortality rates across several causes of death, including malignant neoplasms (any) (24%), intestine and rectum neoplasms (77%), ‘all other’ circulatory diseases (76%) and alcohol-attributable deaths (166%). Excess cancer incidence was also observed in this group for malignant neoplasms (any) (14%), neoplasms of the intestine and rectum (42%), trachea bronchus and lung (40%), alcohol-related (41%) and smoking-related neoplasms (22%), compared with non-Porton Down veterans. There was no evidence of an association between sarin exposure and all other causes of death or types of cancer.

Our study benefits from a large study sample, detailed exposure data and an unusually long follow-up, which has allowed for a novel examination of the relationship between sarin exposure and long-term health. The Porton Down veterans’ cohort is assembled from historical records and national registry data, which helps limit attrition and the potential for selection bias. A limitation of the analysis is that cancer registry data was not available prior to January 1971, which introduces possible survivor bias. We also do not have data on established risk factors for mortality and cancer incidence, including risk-taking and health behaviours (eg, smoking and alcohol consumption) and social disadvantage. We have no evidence that the prevalence of these factors should be higher in sarin-exposed veterans at baseline, as they were matched with unexposed veterans by service number, which approximated the date and location of recruitment. If this were the case, we would expect to see raised hazard ratios across a range of outcomes typically associated with, for example, smoking, alcohol and social disadvantage,^19^ which was not apparent (eg, smoking remains a strong independent risk factor of cardiovascular mortality even at older age^20^). We also know that service personnel were sometimes selected for sarin trials depending on the results of medical screening.^21^ It could be speculated that personnel in better health were more likely to participate in trials, which could potentially bias our estimates towards the null. Finally, we cannot rule out chance findings due to testing multiple exposure–outcome associations; given that our analyses were largely exploratory, our results and corresponding hypotheses require testing in confirmatory studies.

Our study is consistent with our original linkage of the Porton Down Veterans Cohort Study with mortality and cancer incidence up until December 2004.^9 10^ That analysis explored associations between ‘any sarin’ exposure and selected outcomes, adjusting for age and calendar period only but did not account for chemical confounding. Rate ratios (RR) were raised for all-cause mortality (RR=1.09, 95% CI 1.01 to 1.18), diseases of the digestive system (RR=1.30, 95% CI 0.90 to 1.88) and circulatory system (RR=1.15, 95% CI 1.02 to 1.30). Likewise, there was weak evidence of excess cancer incidence in age and calendar period-adjusted associations. In contrast, prior research examining participants of US Military trials demonstrated no excess of all-cause or cause-specific mortality (including cancer and heart disease deaths) in sarin-exposed personnel; however, the US Military trial results are considered largely uninformative due to methodological limitations, for example, the unexposed groups were known to be less healthy.^1 22^ Likewise, most studies of US Gulf War veterans potentially exposed to sarin and cyclosarin during the 1991 Khamisiyah chemical munitions destruction have found little evidence that Khamisiyah-exposed veterans have increased rates of mortality,^15 23^ self^24^ or physician-reported health conditions (including cancers, heart disease and mental disorders)^25^ or clinic and hospital visits^26^ compared with non-Khamisiyah exposed veterans. Discrepancies with our findings could be explained by our longer follow-up, with studies of Gulf War veterans limited by potential exposure misclassification and veterans’ co-exposure to multiple environmental hazards during deployment.

We found a robust association between veterans’ sarin exposure and ‘all other circulatory’ diseases (eg, diseases of arteries, arterioles and capillaries and of veins, lymphatic vessels and lymph nodes, not elsewhere classified), but no associations were evident for ischaemic heart diseases or cerebrovascular diseases. Abnormal ECG and autonomic nervous function tests have been reported in survivors of the confirmed sarin attacks in Tokyo and Matsumoto, Japan (which caused both immediate fatalities and acute symptomatology), but results were normal at 5-year follow-up.^27^ Over a 9-year follow-up, Khamisiyah possibly exposed veterans demonstrated an increased risk of hospitalisations, specifically from cardiac dysrhythmias (RR=1.23, 95% CI 1.04 to 1.44)^28^ but not other events. Mouse studies have shown associations between low-dose sarin exposure and cardiomyopathy and altered stress responses of the hypothalamic-pituitary-adrenal axis, indicating autonomic imbalance^29^ and delayed impairment in heart rate variability,^30^ suggestive of biological plausibility.

The International Agency for Research on Cancer has not classified sarin as to its carcinogenicity to humans due to a paucity of human and animal studies.^2^ In veterans exposed to sarin without physical protection, we demonstrated excess cancer incidence for neoplasms of the ‘intestine and rectum’, ‘trachea, bronchus and lung’ and alcohol and smoking-related neoplasms, compared with non-exposed veterans; results that were largely mirrored in mortality analysis. We found no evidence that sarin-exposed veterans had increased rates of neoplasms of the brain or lymphatic cancers, as suggested in prior nerve agent research.^15 23 31^

Robust associations were observed between sarin exposure and alcohol-related outcomes. Higher alcohol use could potentially be explained by psychological injury through veterans’ involvement in the programme^32 33^ or by a direct physiological effect; for example, there is evidence that sarin exposure is associated with long-term morphological and histological changes to nervous system tissues.^1^ Notably, moderate to heavy alcohol consumption has been associated with a 1.2-fold to 1.5-fold increased risk of cancers of the colon and rectum compared with no alcohol consumption,^34^ which may account for our observed excess in intestine and rectum cancers. A single study reported an excess in gastrointestinal symptoms in Danish Gulf War veterans, but symptoms were not explained by self-reported exposure to nerve agents.^35^

Associations between sarin exposure and alcohol-related deaths and cancers could also be explained by Porton Down veterans’ longer total duration of service,^33^ which is likely associated with being deployed to the theatres of war. Deployment has been associated with an increased risk of alcohol abuse in the Gulf War (pooled OR: 1.33, 95% CI 1.22 to 1.46)^36^ and other veterans; nevertheless, it is unlikely our results are due solely to confounding or selection bias.

In summary, our findings show that the large majority of veterans exposed to sarin at Porton Down did not have increased rates of cancer incidence or mortality, which may act to reassure those who participated in the ‘Service Volunteer Programme’.^9 10 33^ We found no evidence of an association between sarin exposure and brain and other central nervous system cancer incidence or mortality, as suggested in prior research.^23^ A small number of veterans showed an increased rate of some types of cancer, particularly alcohol-related, and higher rates of ‘other’ cardiovascular mortality (ie, excluding ischaemic and cerebrovascular diseases), which may warrant further investigation. For example, the UK Ministry of Defence may seek to update earlier work examining cancer incidence rates and mortality in Gulf War veterans,^37^ which may help to provide much-needed clarity for Gulf veterans. In our study, it is not possible for us to attribute our findings causally to the effects of sarin, chance, systematic bias or a combination of these factors; nevertheless, health providers should be aware of potential health concerns in civilian, emergency responder and military populations who risk potential sarin exposure.

## supplementary material

10.1136/oemed-2024-109525 online supplemental file 1

## Data Availability

Data may be obtained from a third party and are not publicly available.
