# Supplementary material for: Sarin exposure, mortality and cancer incidence in UK military veterans involved in human experiments at Porton Down: 52-year follow-up
Source: Occup Environ Med. 2024 Sep 30;81(9):e109525. doi: 10.1136/oemed-2024-109525 (PMC11503124; doi:10.1136/oemed-2024-109525)
Supplement: online supplemental file 1 [file oemed-81-9-s001.pdf]

Supplementary tables and figures

OEM Archer et al Sarin Exposure, mortality and cancer incidence in UK military veterans involved in human experiments at Porton Down: 52-year follow-up.

Supplementary figure 1. Participant flow

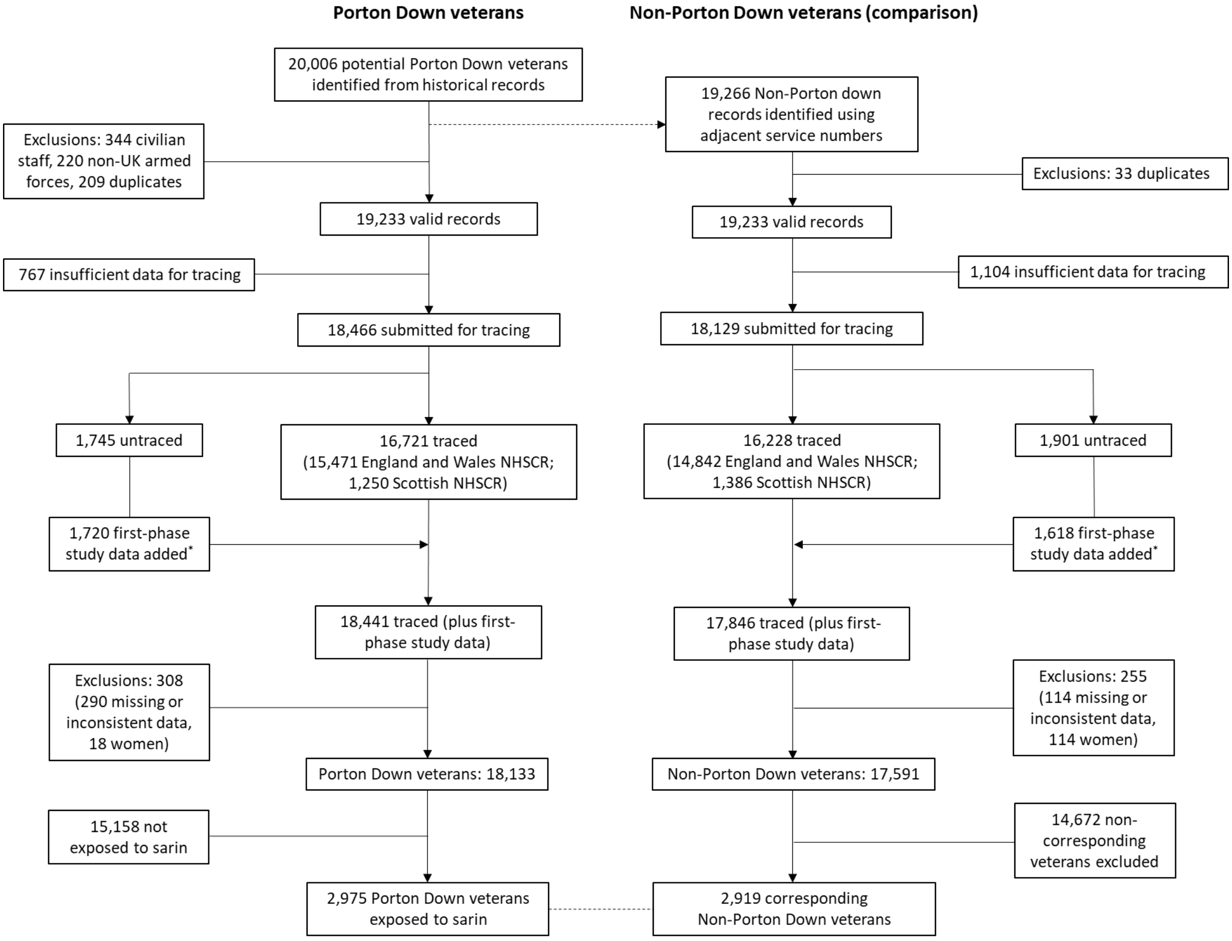

Supplementary table 1. Fully adjusted hazard ratios for the association between veterans exposed to sarin-only (including and excluding co-exposure to rubber mixes and sulfur mustard sensitivity tests) and all-cause mortality, and cancer incidence

	**HR (95% CI) all-cause mortality**		**HR (95% CI) for cancer incidence**	
	**Sarin-only (including rubber mix and sulfur mustard sensitivity)[a]**	**Sarin-only (excluding all other chemicals)[b]**	**Sarin-only (including rubber mix and sulfur mustard sensitivity)[c]**	**Sarin-only (excluding all other chemicals)[d]**
**Sarin exposure**				
(yes/no)	1.08 (1.00, 1.18)	1.10 (0.99,1.22)	1.05 (0.94,1.17)	1.11 (0.97,1.27)
**Number of sarin tests**				
1	1.07 (0.98, 1.17)	1.07 (0.96,1.20)	1.04 (0.93,1.16)	1.11 (0.96,1.28)
2 or more	1.26 (1.00, 1.59)	1.30 (1.01,1.66)	1.04 (0.76,1.43)	1.11 (0.78,1.57)
**Exposure quantity**				
Unknown	1.13 (1.01, 1.27)	1.18 (1.02,1.37)	1.06 (0.91,1.24)	1.06 (0.87,1.30)
Low (Air <15.0 mg min/m^3)	1.03 (0.92, 1.15)	1.01 (0.87,1.16)	1.01 (0.88,1.16)	1.15 (0.96,1.38)
High (Air $\geq$15.0 mg min/m^3)	1.15 (0.94, 1.41)	1.17 (0.88,1.57)	1.07 (0.83,1.38)	1.13 (0.79,1.61)
**State[e]**				
Vapour	1.08 (0.98,1.18)	1.08 (0.96,1.21)	1.05 (0.93,1.18)	1.14 (0.99,1.32)
Liquid	1.11 (0.96,1.29)	1.21 (0.97,1.52)	1.01 (0.83,1.23)	0.96 (0.70,1.31)
**Physical protection**				
No	1.11 (1.01, 1.23)	1.10 (0.97,1.26)	1.12 (0.99,1.26)	1.19 (1.01,1.40)
Yes	1.03 (0.91, 1.17)	1.09 (0.93,1.27)	0.89 (0.75,1.06)	0.98 (0.80,1.21)
**Chemical protection**				
No	1.08 (0.99, 1.19)	1.10 (0.98,1.24)	1.04 (0.92,1.17)	1.09 (0.94,1.26)
Yes	1.10 (0.93, 1.29)	1.08 (0.87,1.32)	1.05 (0.85,1.29)	1.19 (0.92,1.55)

Models adjusted for age, calendar period, branch of service, previous duration of service, and place of birth

a: Estimates based on 1,430 veterans exposed to sarin-only (allowing for co-exposure to rubber mixes and mustard sensitivity tests)

b: Estimates based on 756 veterans exposed to sarin-only (excluding all other chemicals)

c: Estimates based on 1,385 veterans exposed to sarin-only (allowing for co-exposure to rubber mixes and mustard sensitivity tests)

d: Estimates based on 734 veterans exposed to sarin-only (excluding all other chemicals)

e: Excludes 47 veterans with missing state data
